# Towards exergaming commons: composing the exergame ontology for publishing open game data

**DOI:** 10.1186/s13326-016-0046-4

**Published:** 2016-02-09

**Authors:** Giorgos Bamparopoulos, Evdokimos Konstantinidis, Charalampos Bratsas, Panagiotis D. Bamidis

**Affiliations:** Medical Physics Laboratory, Medical School, Faculty of Health Sciences, Aristotle University of Thessaloniki, Thessaloniki, Greece; Mathematics Department, Aristotle University of Thessaloniki, Thessaloniki, Greece

**Keywords:** Serious games, Exergames, Ontology, Linked open data, Exergame commons, Active and healthy ageing, Open clinical trials

## Abstract

**Background:**

It has been shown that exergames have multiple benefits for physical, mental and cognitive health. Only recently, however, researchers have started considering them as health monitoring tools, through collection and analysis of game metrics data. In light of this and initiatives like the Quantified Self, there is an emerging need to open the data produced by health games and their associated metrics in order for them to be evaluated by the research community in an attempt to quantify their potential health, cognitive and physiological benefits.

**Methods:**

We have developed an ontology that describes exergames using the Web Ontology Language (OWL); it is available at http://purl.org/net/exergame/ns#. After an investigation of key components of exergames, relevant ontologies were incorporated, while necessary classes and properties were defined to model these components. A JavaScript framework was also developed in order to apply the ontology to online exergames. Finally, a SPARQL Endpoint is provided to enable open data access to potential clients through the web.

**Results:**

Exergame components include details for players, game sessions, as well as, data produced during these game-playing sessions. The description of the game includes elements such as goals, game controllers and presentation hardware used; what is more, concepts from already existing ontologies are reused/repurposed. Game sessions include information related to the player, the date and venue where the game was played, as well as, the results/scores that were produced/achieved. These games are subsequently played by 14 users in multiple game sessions and the results derived from these sessions are published in a triplestore as open data.

**Conclusions:**

We model concepts related to exergames by providing a standardized structure for reference and comparison. This is the first work that publishes data from actual exergame sessions on the web, facilitating the integration and analysis of the data, while allowing open data access through the web in an effort to enable the concept of Open Trials for Active and Healthy Ageing.

## Introduction

Continuous monitoring through self-tracking tools such as serious games may result in better health assessments. These games produce large amounts of data, however, if they are published in a non-standardized way , the capacity to exploit data richness, by combing and integrating patient-related datasets will be destroyed, which may weaken its assessment capabilities. Moreover, large volumes of data are nowadays collected from applications that monitor posture and body movements. The difficulty of assessing physical activity and/or sedentary behavior can be addressed by describing the data in a standardized format. A recent study has presented the ontology of physical activity (OPA) in order to provide a formal description of physical activity [1]. The ontology of physical exercise (OPE) was developed towards a similar purpose, in an attempt to describe the physical exercise in terms of functional movements, muscles involved, as well as, hardware and monitoring devices that are used [2].

In recent years, there has been an increased interest by the game industry in game controllers that enable user interaction through body movements such as Wii Balance Board and Microsoft Kinect. These technological advances have led to the development of the so called exergames [3], a special kind of serious games, which combine gaming and exercise resulting in maintenance and improvement of physical status, focusing on large muscle groups. Serious games in general have an impact on users such as a change in knowledge, behavior, physical state, cognitive function as well as health and mental well-being [4]. Moreover, they may form a part of the health care system in the foreseeable future, as they enable focusing on the patient needs through personalized interventions demanded by recent focus on active and healthy ageing [4]. Konstantinidis et al. [5] presented an exergaming platform which was used in the Long Lasting Memories project [6–8]. In the latter papers, it was implied that the perceived, by the elderly users, usefulness of the physical exercise through exergames contributes to their motivation and their subsequent adherence to the exercise protocol. In accordance with other studies, it was also reported that exergames can positively impact many health areas, such as balance, gait [9], motion control [10], quality of life [5], mood and sociability [11, 12], self-esteem and reduced risk for depression [13]. Regular exercise at home could help to prevent diseases aggravated by a sedentary life imposed by conditions such as cardiovascular diseases and stroke [14]. Furthermore, health benefits from exercises could contribute to the reduction of health care costs for insurance companies and the public health system as well [15].

Only recently some studies started investigating and presenting exergames as health monitoring tools, through collection and analysis of the data produced by their metrics [16]. Such metrics are quantitative measures of the characteristics of game objects, used to record a player’s performance and behavior. They range from simple measures such as rating and total game time to more complex ones, which are calculated by combining various variables/features [4]. More specifically, metrics of balance games have been studied in the context of fall prediction [17, 18], while other studies have found significant correlations between response time and the risk of falling [19]. Besides this, measurements such as grip strength, heart rate, weight and speed, measured during exergaming sessions, have been proposed as potential indexes of frailty [20]. The use of game metrics as monitoring and assessment tools is followed by low cost development, time saving [16] and real time recording and user’s performance visualization of [15, 16]. Finally, game metrics are considered as reliable determinants towards exploitation for personalized game difficulty adjustment [21, 22].

Moreover, the unobtrusive monitoring attitude of games contributes to elimination of user anxiety which is apparent in conventional clinical cognitive assessment, as users do not realize the fact that they are being tested; they are, therefore, absorbed by the game [23]. According to literature, assessments performed using game metrics and algorithms could be equally or even more effective and reliable than those performed in clinical settings [15, 20]. Furthermore, the role of the serious games’ in-game metrics in early detection of cognitive or physical decline symptoms has recently gained the interest of researchers [24–26].

A point that deserves attention, however, is the fact that the lack of experience in using new technologies can cause high mental and emotional stress as well as cognitive overload. Moreover, it is difficult to correlate user interactivity in a game with game metrics as well as with clinical outcomes, since any two users playing the same game may indeed have pretty different experiences [4].

However, understanding and further exploiting game data derived from game metrics can be based on standards and knowledge sharing, among people involved in the design and development of exergames, as well as, its user deployment [15].

The majority of existing games are designed for recreational purposes aimed at a typical healthy person, making it particularly challenging for elderly users [5]. Therefore, the development of exergames that target specific populations [27, 28] such as people with dementia or cognitive decline and memory or vision impairments is of paramount importance [15, 29]. In light of this, some researchers have developed user-tailored games by combining game data with physical exercise protocols [30] (planned, structured and repetitive motion in order to maintain or improve physical fitness and body capacity [31]) or even just physical activity (body movement produced by the contraction of skeletal muscles).

Following this concept, a noteworthy number of ontologies covering different domains of this type of serious games have emerged over the last years. Ontologies describing games and gameplay achievements [32], game development [33], multiplayer entities [34], graphics [35], physical activity [1] and physical exercise [2] are publicly available. However, the fact that exergames have just started being considered as “therapeutic” interventions [15, 36], along with the immature exploitation of their associated performance metrics, calls for an exergame ontology combining all the relevant ontologies and further developing and describing recently introduced concepts. Needless to say that the number of papers presenting game metrics as a tool of cognitive and physical assessment in correlation with clinical assessment tests is rather limited. The current piece of work presents the first ontology that describes exergames and puts emphasis on detailed performance metrics. Built on top of others, after a scrutiny of the exergame literature, the proposed ontology focuses on the semantic description of games and game sessions and combines the concepts of game and exercise. More specifically, the ontology introduces non-gaming concepts such as physical exercise, muscles involved and necessary equipment for games. This study suggests a unified model for concepts related to exergames. To that extent, it introduces a well-structured model for exergames description and open game metrics data publishing, thereby incorporating game-related concepts such as game components, sessions and results. Consequently, it aims to facilitate monitoring and understanding of health-related situations though the establishment of common vocabularies and data exchange standards through exergames. The proposed ontology was adopted and integrated in an exergaming platform which was then piloted by fourteen (14) elderly users who participated in a number of game sessions. The acquired game results were automatically converted to RDF triples and published on the web as open data, accessible through a SPARQL Endpoint.

The remainder of this paper is structured as follows. The background section enumerates the existing ontologies in the domain of games as well as some exergaming paradigms that already highlight the value of their in-game metrics and performance indicators. In the methods section the development tools and processes are presented. The results section presents details of key concepts composing the exergame ontology. An exergaming platform realizing and utilizing the proposed ontology is demonstrated in the same section along with information on accessing the RDF triples, derived from the actual game sessions, through a SPARQL endpoint. At the end of the paper light is shed on the importance of this work towards contributing to healthcare thereby putting emphasis on monitoring and assessing the player’s status. Finally, the research limitations and challenges of this work are discussed along with further envisaged work.

## Background

Similarly to the need for describing diseases in standardized ways by using suitable vocabularies such as International Classification of Diseases (ICD) [37] and Systematized Nomenclature of Medicine (SNOMED) [38], there seems to be an emerging need to allow standardized descriptions of user interactions with exergames. An early effort along these lines was the Game Ontology Project (GOP) [32], which was one of the first attempts toward the semantic description of games; it provides a structured model for the game and establishes relationships among its elements. GOP consists of five main components, namely, the user interface, rules, goals, entities and entity manipulations. As far as game metrics are concerned, GOP includes metrics such as score, time and success level. The Game Content Model (GCM) is another ontology developed for serious games aiming at game development facilitation by non-specialists in the context of role-playing and simulation games [33]. GCM creators have spotted that the GOP described game concepts from the end user perspective and did not include notions that describe the game environment, its structure and its events. Thus, Chan and Yuen [39], based on the GOP, developed the Digital Game Ontology (DGO) which expressed events and concepts related to games, such as user’s actions and production details, while they used the Semantic Web Rule Language (SWRL) to model game rules. They also highlighted the need for a standard format for data recorded by the game since that could contribute to the understanding of user-game interaction. Moreover, Mepham and Gardner [34] developed an ontology for online multiplayer games; this ontology described the common elements of games such as players and game objects. Roman et al. [40] suggested an ontology for role-playing games, including elements such as characters, story and resources. Furthermore, an ontology consisting of three main components comprised of graphics, configuration entities and multimedia has been presented in the domain of mobile video games [35].

Semantic web technologies can transform games from isolated standalone programs to distributed and interconnected systems enabling the combination of user data with other datasets to produce useful knowledge [34]. Moreover, the use of ontologies and SWRL rules modelling could be deemed as an artificial intelligence methodology [40]. Furthermore, game ontologies provide a structured vocabulary that facilitates modelling and representation of design specifications as well as maintenance and development of games [41]. In contemporary projects with large groups from all over the world, knowledge management, which can be further facilitated by game and other semantics web technologies [42, 43], could be vital in game development.

According to recent literature, there are no available methods established for evaluating the data generated by game metrics [44]. Recently, exergames were attributed a role in unobtrusive health monitoring. However, the evaluation of game algorithms, such as those calculating expenditure and heart rate through game controllers or assessing physical and cognitive status, requires researchers to compare the data generated by these games with measurements from calibrated and certified devices (calorimeters, heart rate monitors, etc) or clinically valid assessment tests. For instance, studies have shown that calculation of energy expenditure through games has moderate correlation with corresponding measurements from external devices [16], while game metrics have moderate correlation with cognitive assessment tests [32].

However, in a typical scenario nowadays, game data are usually the very property of gaming companies thereby resulting in inability to verify any potential clinical outcomes of serious games. If only exergaming metrics were proved reliable and valid, exergames could replace external devices or even ideally account as additional measurement methods and information providers [16]. It is, therefore, imperative that there is a need to “open” this kind of data. One could appreciate that the incorporation of common models, specifications and data interoperability standards through a commons approach may result in a more effective collaboration among researchers and assist in the design and development of such games as well as knowledge sharing. Exergame commons, comprising shared knowledge, standards and tools, along with other initiatives such as open data commons [45] which may facilitate publishing of game metrics data, would promote collaborative and community-driven research thereby maximizing the return on investment [46].

## Methods

### Ontology editing tools and resources

The ontology was implemented using standard semantic web technologies namely the Resource Description Framework (RDF), RDF Schema (RDFS) and Web Ontology Language (OWL), while some parts of the ontology were developed using Protégé Desktop 5.0 beta ontology editor [47]. Protégé is an open-source platform that provides tools to develop ontologies and knowledge-based applications.

### Ontology development process

During the design and development process, we followed the steps outlined in the work of Noy & McGuinness [48]. Firstly, the field of exergames as well as the potentials uses of the ontology were carefully examined. The author team has been designing and developing exergames for more than seven years so far. Such games have been tested in large-scale pilots with over 200 participants, facilitating a deep understanding of player-game interactions [5, 36]. Extended analysis of data has been conducted deriving different game metrics, but also revealing their likely correlations with clinical assessment tools. These findings led them also to suggest some exergaming design guidelines for stealth assessment of cognitive and physical status [49]. In addition, the authors have already investigated the contribution of the exergame results to decision support systems [50] and the use of new, accessible to seniors technologies for the development of exergames [51]. In order to obtain a deep understanding of all aspects and concrete entities comprising exergames themselves, as well as, exergame sessions, the author team has organized meetings, where multidisciplinary researchers such as exergame developers, psychologists and physical trainers/sport scientists, have attempted to collaboratively define key exergame elements to be recorded. Then, a detailed search in literature as well as in repositories of biomedical ontologies such as BioPortal [52], Ontobee [53] and AberOWL [54] was performed in order to find relevant ontologies. Various relevant ontologies were identified both in research papers as well as in biomedical repositories. The most suitable ones were incorporated in the proposed exergame ontology: i) FOAF: this ontology is used for the description of players since it is one of the most popular ontologies for the representation of people profiles; ii) OPE: this ontology was found at then BioPortal repository and is used for the representation of physical exercises. The exergame ontology utilizes OPE for the semantic description of the exercises involved in games; iii) NCI Thesaurus (NCIt) for the description of muscles involved in exercises: this ontology was found at the BioPortal repository and is used for the description of muscles that are not included in OPE; iv) “Quantity, Unit, Dimension and Type” collection of ontologies (QUDT) for defining the units of measuring of game metrics; such metrics are used in measuring of physiological variables like heart rate and blood pressure, while others focus on physical dimensions such as time, degrees, etc. To be of utility when analyzing the performance of the person doing the exercise, these metrics have both value and unit of measurement. QUDT was selected for the expression of the measurements; v) vCard for information about the site where a game session is carried out and vi) SKOS for the development of a vocabulary of common terms for exergames. Afterwards, a list of game-related concepts was developed and the class hierarchy was defined. Since there are many ways to develop an ontology and model the concepts related to a particular domain, we have defined a straightforward class hierarchy, aiming to provide an easy-to-use model which can be effortlessly integrated to exergames. Subsequently, the properties that represent relationships between classes were identified and the property restrictions were defined. In this step, difficulties encountered linked with game metrics representation, since it was needed to model an n-array relationship. For this reason, an auxiliary class was developed to associate game results, metrics and their values. Moreover, in order to let end-users have several options when it comes to property ranges, restrictions were kept to a minimum. Finally, instances of classes were developed, which facilitate the connection of different data sets by referencing to same concepts. The development of the ontology was a repetitive process where the multidisciplinary team contributed to each stage.

### Availability

The developed exergame ontology is available through the OCLC Persistent uniform resource locator (PURL) http://purl.org/net/exergame/ns#. This Uniform Resource Identifier (URI) is dereferenceable, serving both RDF document and ontology documentation in HTML format, depending on the request. The documentation is generated automatically using the Live OWL Documentation Environment (LODE) [55]. In oder to facilitate the usage of the ontology, apart from the RDF/XML format of the ontology, this methodology enables end-users to retrieve a description of the resource through their browser at the same URI, in line with repositories of biomedical ontologies such as Ontobee and BioPortal providing an interface for all ontology terms.

## Results

**Fig. 1:**
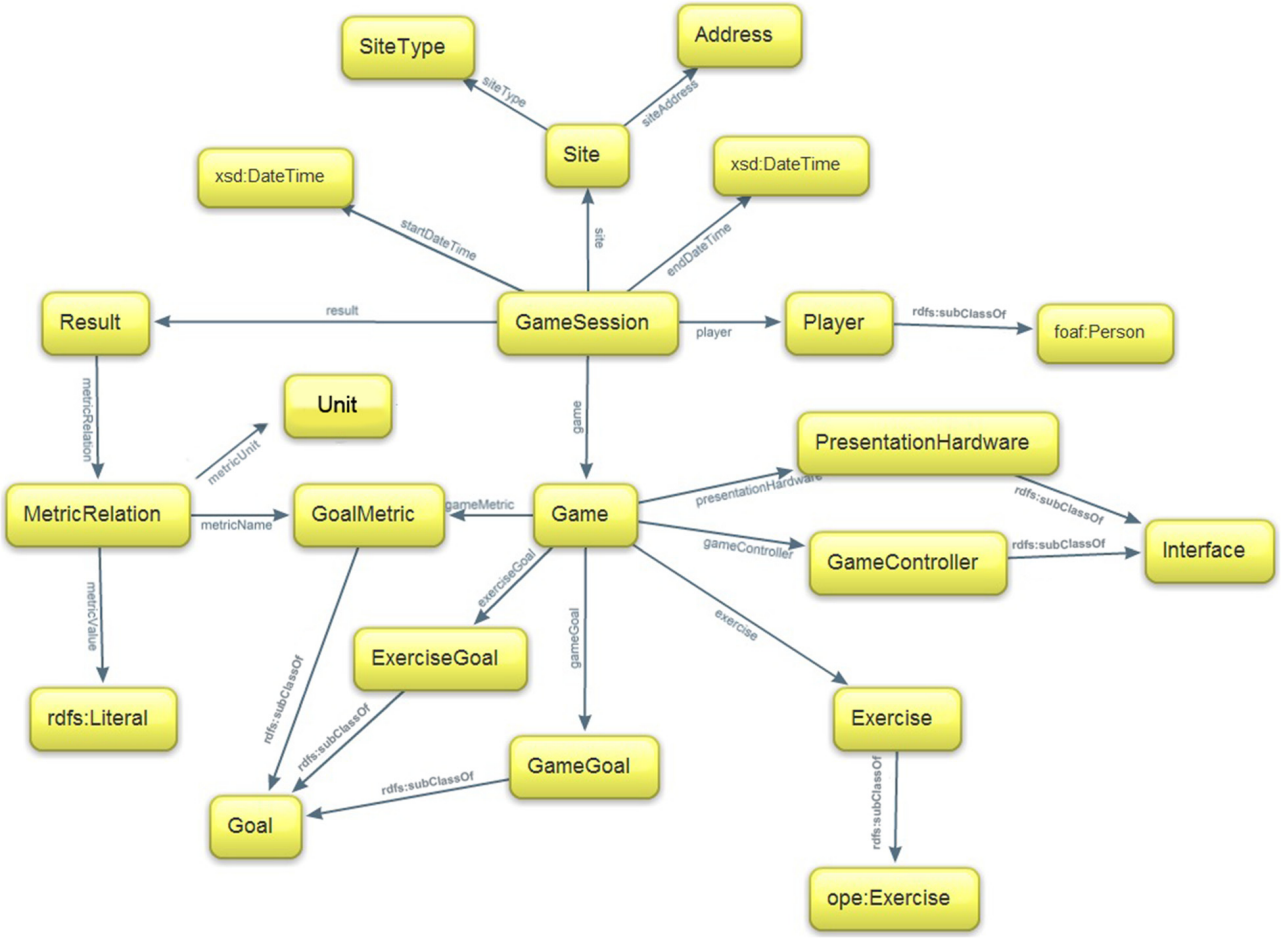
Exergame ontology overview. A graphical representation of the ontology. All classes and properties that do not have a prefix belong to exergame ontology

The purpose of this study is to investigate and provide a structured description of the key concepts related to exergames such as components of games, game sessions and game results, composing the exergame ontology. Since the nature of the data produced by different games were highly diverse and heterogeneous, special attention was given to the design of the structure of game results towards a universal model for all exergames. All classes and properties of the exergame ontology are depicted in Fig. 1.

**Fig. 2:**
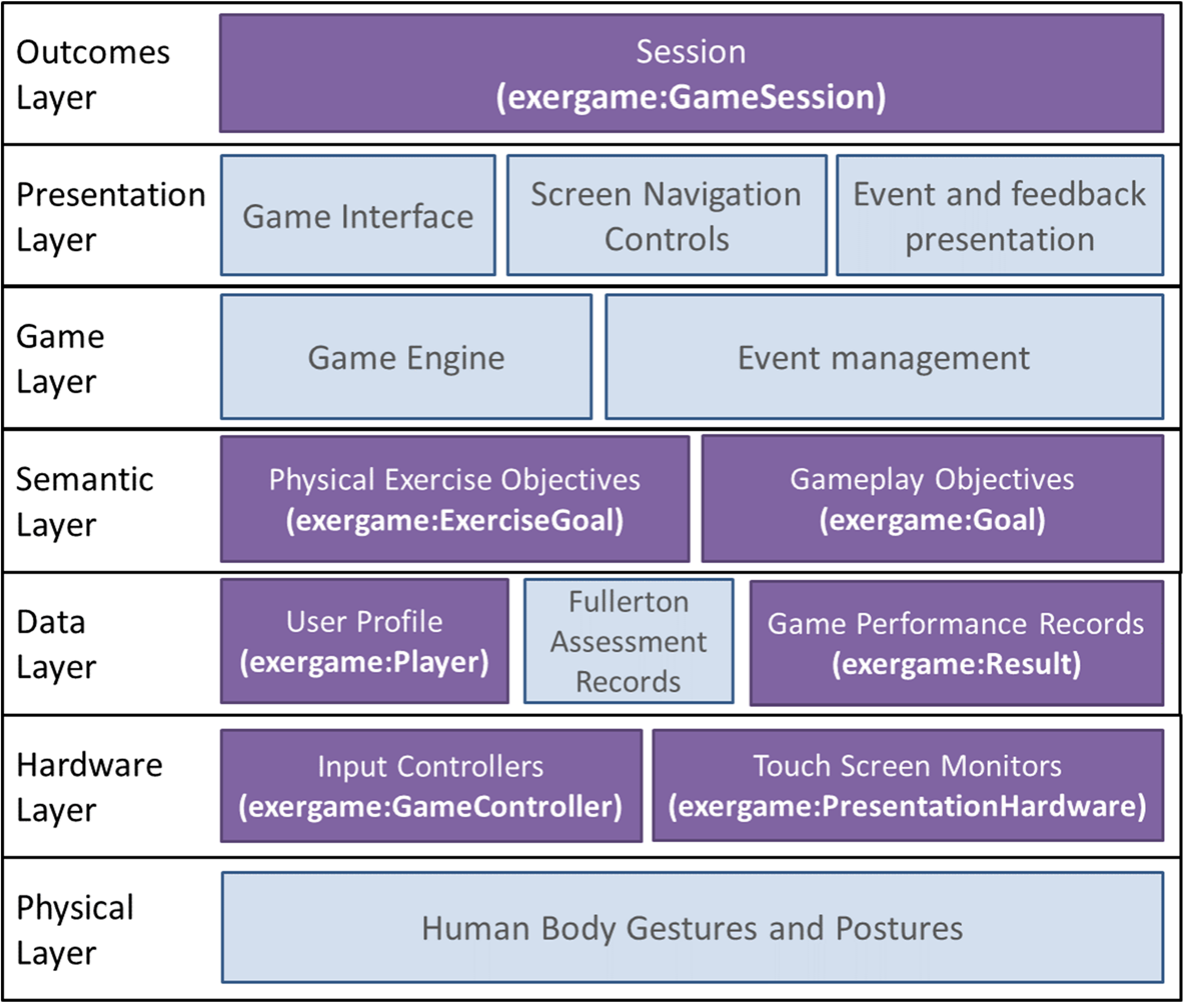
Exergame architecture. An illustration of exergame’s components along with their semantic description. Dark blocks refer to concepts that can be modeled using the exergame ontology. In each of these blocks, the classes being used to model these concepts are included inside brackets. The remaining (light) blocks will be incorporated to the ontology in a following version.

Konstantinidis et al. [5], proposed an architecture scheme of exergames, by defining the fundamental building blocks of an exergame. Aligning with their paradigm, the exergame ontology incorporates classes that refer to most of the layers and components of that architecture (Fig. 2). In this study, the intention was to cover exergames from a user monitoring point of view, thereby focusing on game sessions, players and game results. At a later stage, further game-related components would be incorporated in the ontology, such as the game engines used for its development as well as event management details.

In the sections below, each concept of the developed ontology is presented separately.

### Game

For the description of a game as an entity we use concepts from GOP and GCM including goals, game controllers and presentation hardware. However, taking into account the particularities of exergames, the ontologies were extended to cover new concepts such as physical exercise and auxiliary equipment (e.g. weights and fitness springs). For each game, an instance of class exergame:Game is created and then all sessions refer to this game by its URI.

### Goals

This section includes game goals, exercise goals and game metrics. Game goals refer to the objectives and conditions that must be fulfilled in order for the player to succeed in the game. They could be either high level objectives such as victory or success at some level, or lower level objectives such as avoiding an obstacle. As each game incorporates a physical exercise, one of the main objectives is the proper execution of this exercise which is referred to as an “exercise goal”. Game metrics are shared among the games and thus instances of the class exergame:GoalMetric, that could be referenced by all games, should be developed. A list of instances which was developed in this study is available at http://purl.org/net/exergame/metric#. Moreover, each game metric should have a unit of measurement which is an instance of the class exergame:Unit, an equivalent class of qudt:Unit. Some instances of the qudt:Unit can be found at the QUDT Units Ontology [56].

### Player

Refers to the user playing the game and it is connected with game sessions and their results. The FOAF ontology is used to describe each player through the foaf:Person class.

### Interface

Interface is the shared boundary between user and computer and refers to how the player interacts with the game as well as how the game is presented to the player. It consists of the presentation hardware and the game controllers. The former represents the device on which the game is presented (e.g. tablet, computer, smart phone, video game console, etc.). The latter stands for the devices which provide input to a game in order to control an object or a character in it (e.g. keyboard, joystick, steering wheel, etc.). The game controllers include also contemporary controllers which detect motion and user’s postures and gestures (e.g. Kinect, Wii Balance Board and Wii Remote) as well as other recently introduced devices which record brain activity such as NeuroSky MindWave and Emotiv. Instances of the class exergame:GameController were developed for most modern game controllers and are available at http://purl.org/net/exergame/controller#. These instances were connected through the owl:sameAs property with the corresponding resources of DBpedia, which is the core of linked data. Due to the small size of game controllers alongside the low rate of new emerged controllers, the links between these instances and DBpedia were done manually, since the use of automatic tools was not worth the effort. If the number of instances increases dramatically, employing automatic and semi-automatic semantic linkage and integration frameworks such as SILK [57] will be of paramount importance.

### Physical exercise

Each exercise is described using the OPE ontology including the muscles involved, the equipment used during the exercise as well as possible health benefits, like improved fitness and clinical outcomes. Physical exercises like “biceps” and “leg extension” are represented as instances of the class exergame:Exercise.

### Game session

Game session refers to the period in which a player is playing a game. It begins when the game starts and ends either with the end of the game or when for some reason the game stops. The session, apart from the start and end time, is associated with concepts like the player, the game itself, the site and the data produced by game metrics.

### Game results

A game result includes all data derived from game metrics in one session of a game. Each game may have more than one metrics and more than one iterations (referring to the corresponding physical exercise iteration). Each game session is associated with an instance of the class exergame:Result and this instance is linked with a set of game metrics along with their values and units of measurement. In RDF and OWL, a property is a binary relation used to link two instances or an instance to a literal value. For this reason, in order to define an n-array relationship [58] that includes an instance of the class exergame:Result, instances of the classes exergame:GoalMetric and exergame:Unit as well as an RDF literal which represents the metric’s value, the auxiliary intermediate class exergame:MetricRelation is used, which represents this relationship. In case that a game consists of one metric and iteration, the session’s result includes an instance of exergame:MetricRelation which is linked to a metric, a unit of measurement and a value. In the event of more iterations, the property exergame:metricValue links to a sequence of metric values using rdf:Seq.

### Site

The site refers to the venue where a game is conducted including the address and the type of it (e.g. “Ecologically valid Active and Healthy Ageing Living Lab area - Medical Physics Laboratory of the Aristotle University of Thessaloniki”). Venue details may include street address, email address, telephone numbers, coordinates etc. For these details, many well-known ontologies could be used such as vCard, but the range of the property “exergame:siteAddress” was left open thereby allowing for any other form. For example, a vCard instance can be used as its value and besides address it can provide site coordinates in the form of geographical coordinates (geo URI) using vCard:hasgeo property. As far as site category is concerned, instances of the class “exergame:SiteType” were developed, which represent specific categories such as home, research laboratory and senior center (http://purl.org/net/exergame/sitetype#).

### Vocabulary for common concepts

To date, there is no common vocabulary for terms related to exergames that uses semantic web technologies. Therefore, in order to support the reuse of exergame concepts, the exergame ontology incorporates the exergame:concept property which links instances to the concept they represent. Moreover, a vocabulary of such terms was developed using the SKOS ontology; this is now available at http://purl.org/net/exergame/concept#. For example, in the case that an exergame includes a time-related metric such as reaction time, the instance that represents this metric could connect with the concept of time, which is an instance of skos:Concept. This vocabulary is intended to be scalable and form an integral part of exergame commons, since it can be viewed as a more general dictionary, just to cover for those researchers for which the already defined concepts do not fit their own needs. As an illustration, when an instance of a class (e.g. an instance that represents a game metric which calculates the distance covered by the player) concerns a concrete concept which is not included in the aforementioned vocabulary, it can be extended by developing an instance of skos:Concept. Therefore, the existence of a rich vocabulary is on the exergame community’s best interest since it facilitates the linkage of two distinct instances that refer to a common concept. As a result, game results derived from distinct exergames can be analyzed together or compared to each other, based on their common characteristics.

### Applications

This study provides opportunities to capitalize on open exergame data for active and healthy ageing. Publishing game results as open data will enable the exergame community to test these data, validate their algorithms, combine game results with other datasets and produce new knowledge. The task of adapting the ontology to an exergame is pretty simple, regardless the technology used; In fact, each game should be semantically described only once. Regarding game sessions, all information, including game results, can effortlessly be semantically enriched and stored to a triplestore as well. On the other hand, in most games the results are stored in a database. Hence, already stored game results can be converted to RDF utilizing a language for expressing mappings from relational databases to RDF datasets, such as R2RML [59]. As far as privacy is concerned, any information about patients that might reveal their identity should not be stored. For this reason, a plain unique identifier that corresponds to a particular patient may be attached to game session’s data.

### Integration in HTML5 exergames

Exergame ontology was integrated in HTML5 games on webFitForAll (wFFA) web platform (Konstantinidis EI, Bamparopoulos G, Bamidis PD: Moving Real Exergaming Engines on the Web: The webFitForAll case study in an active and healthy ageing living lab environment, submitted.). wFFA is an exergaming platform developed to support e-health applications incorporating various game controllers such as Wii Balanceboard, Wii Remote, Microsoft Kinect and NeuroSky MindWave through the transparent Controller Application Communication (CAC) Framework [60]. wFFA contributes to fitness maintenance and well-being of elderly people through specifically designed games/interfaces with simple graphics, which belong to three categories. In the first category, there are games that incorporate physical exercises such as stretching, resistance and weight lifting, aiming at power increase of the upper and lower limbs [5]. These games comprise a predetermined number of iterations and include metrics such as success, reaction time and goal time. Moreover, there are games such as “Apple”, “Hiking”, “Fishing” and “Golf” which are related to physical activities in conjunction with light cognitive tasks, such as walking, balance and reaction. The metrics of these games are total time and player’s score, which is calculated differently for each game (number of apples in the basket, number of steps). Finally, the last category includes interfaces which facilitate the health measurements collection, including but not limited to weight and blood pressure. These measures are treated in the same way as game metrics.

#### Integration architecture

The wFFA is based on a JavaScript library which is responsible for several tasks such as coordination of games and exercise protocols and consists of three main components: the initializing component, the processing component and the output component. For the purposes of this study, specific extensions were developed for each component in order to support the semantic description of players, games and sessions as well as to enable games’ data storage to a triplestore. One of the main tasks of the initializing component is the game controllers’ management and the exercises difficulty adjustment. In this study, an extension was developed that initializes game metrics in order to record player’s behavior.

The processing component accounts for the proper function of the game and consists of a set of subcomponents, being executed during a game session, including common game tasks such as feedback control. We extended this component in order to enable data collection from game metrics resulting in a detailed record of user’s actions. Furthermore a preprocessing component was developed to gather all information about the game session by utilizing the user’s unique identifier to retrieve the URI of the instance of class exergame:Player that represents them, which was stored automatically when the user’s account was created. Any information that could potentially disclose the user identity is omitted. As far as the site is concerned, an HTML form was developed extending the user’s settings to include information about the site (a set of predefined site categories are provided as options). Once this form is completed, site details are added to the session. Finally, the start date and time are added to the session, which is almost ready to be saved.

The output component is activated at the end of each game and is responsible for storing the result of a game session in a relational MySQL database (through a Rest API). In this study, a subcomponent was developed that processes the data from game metrics, describes them semantically according to the ontology and adds them in the game session along with the end date and time. As long as all the information about the game session is converted to RDF triples, it is uploaded in a Sesame triplestore using Ajax. All triples concerning games, exercises, game sessions and patients are stored in a single graph in the triplestore.

#### Description of game “SideRaises”

In the “SideRaises” game, players start by holding hand weights straight down at their sides. Then they have to raise both arms to the side at shoulder height and after they hold this position for a few seconds, they lower their hands and continue with the next iteration. Depending on their physical condition, users can use different weights. The game consists of the following metrics: success, reaction time and goal time and it utilizes a Kinect as a game controller and a smart TV as a presentation hardware. The corresponding exercise involves the deltoid, the infraspinatus, teres minor and major and the latissimus dorsi muscles. For this example, the following prefixes are used:

- @prefix : <http://www.fitforall.gr/resources##>.
- @prefix rdf: <http://www.w3.org/1999/02/22-rdf-syntax-ns##>.
- @prefix rdfs: <http://www.w3.org/2000/01/rdf-schema##>.
- @prefix exergame: <http://purl.com/net/exergame/ns##.
- @prefix exergame-metric: <http://purl.org/net/exergame/metric##>.
- @prefix exergame-gamecontroller: <http://purl.org/net/exergame/controller#http://purl.org/net/exergame/controller##>.
- @prefix ope: <http://www.semanticweb.org/ontologies/2013/2/OPE.owl##>.
- @prefix nci: <http://ncicb.nci.nih.gov/xml/owl/EVS/Thesaurus.owl##>.

The exercise and muscles involved are described first. In the case that a muscle is not involved in OPE, it is defined as a subclass of nci:Muscle.



Thereafter, the :SideraisesGame is defined alongside its metrics, game controller and presentation hardware:



#### Piloting with users in a living lab

A number of elderly volunteers were visiting a specially designed environment in the laboratory of Medical Physics at Aristotle University of Thessaloniki [60] daily and were playing a set of exergames. This environment, facilitating also the trials of USEFIL project [61–65], had two separated areas, a furnished one with sofa, table and chairs to resemble a small living room and an area with a sink that resembles a bathroom. This configuration was designed to create an ecologically valid experimentation environment.

#### Participants

Fourteen elderly people (3 males and 11 females, average age 73.4 years old) who had no previous experience with technology participated in this study. On average, each elderly person visited the specially designed environment for 6 days in total and played/interacted with 7 different games each time. The total time of all game sessions for all users was about 35 h.

#### Game data

The game metrics data which was produced during sessions of the aforementioned games were converted to RDF by the game framework and were uploaded on a triplestore as open data. In order to save the results of game sessions, there are about 30,000 records in the triplestore corresponding to 35 h of gaming. All data are accessible from a SPARQL endpoint that is available at http://www.fitforall.gr/sparql, where queries can be made using the GET or POST method. In Table 1, data from a game session of “SideRaises” game are depicted. For instance, the SPARQL query for getting all values from the metric that measures goal time (exergame-metric:GoalTime) during the 3^rd^ iteration of “SideRaises” game, along with the corresponding player and date of game session, is the following:

**Table 1:** RDF triples produced during a session of “SideRaises” game

Subject	Predicate	Object
^a^_:SideRaisesGameSession	rdf:type	exergame:GameSession
_:SideRaisesGameSession	exergame:result	_: SideRaisesGameResult
_:SideRaisesGameSession	exergame:site	<http://www.fitforall.gr/resources/MedicalPhysicsLaboratoryAuth>
_:SideRaisesGameSession	exergame:startDateTime	2014-09-22T18:09:39 02:00
_:SideRaisesGameSession	exergame:endDateTime	2014-09-22T18:09:39 02:00
_:SideRaisesGameSession	exergame:player	<http://www.fitforall.gr/resources/player162>
_:SideRaisesGameSession	exergame:game	<http://www.fitforall.gr/resources/SideRaisesGame>
_:SideRaisesGameResult	rdf:type	exergame:Result
_:SideRaisesGameResult	exergame:metricRelation	_:MetricRelation1
_:SideRaisesGameResult	exergame:metricRelation	_:MetricRelation2
_:SideRaisesGameResult	exergame:metricRelation	_:MetricRelation3
_:MetricRelation1	exergame:metricName	<http://purl.com/net/exergame/metric## Success>
_:MetricRelation1	exergame:metricValue	_:node197jv5qonx686
_:node197jv5qonx686	rdf:type	rdf:Seq
_:node197jv5qonx686	rdf:_1	TRUE
_:node197jv5qonx686	rdf:_2	TRUE
_:node197jv5qonx686	rdf:_3	FALSE
_:node197jv5qonx686	rdf:_4	TRUE
_:node197jv5qonx686	rdf:_5	FALSE
_:node197jv5qonx686	rdf:_6	TRUE

^a^For brevity, _:genid-6569e0b8e8774a22b73a515a98c0f963- is abbreviated as _:

## Discussion

In this study, an ontology for exergames was developed by fusing concepts from gaming and physical exercise into a single structure. As existing gaming ontologies target mainly at the game design and development including concepts such as game scenario and game objects [32, 33, 39], they do not provide a comprehensive model that describes the data produced in a game session. As a consequence, a standard structure that facilitates publishing of game data on the web was developed. This was achieved by modelling the concept of the game session, which in combination with the game, the player and the game results, formed a universal model for the semantic description of the playing process. This structure could then be effortlessly integrated into other existing ontologies.

Another neat contribution of this work though, is that of opening the game data emerging from the aforementioned game sessions. For the first time, such data were described semantically in a machine-readable format using W3C standards and some instances are linked to related resources from DBpedia. Complying with the highest level in the 5-star open data scheme introduced by Tim Berners-Lee [66], this dataset is, to the best of the authors’ knowledge, the world’s first available open dataset of exergames metrics described semantically by means of Linked Open Data (LOD). Furthermore, opening game data in an RDF format is in researchers’, caregivers’ and patients’ best interests, as game analytics and data visualization techniques can be subsequently applied, enabling them to monitor patients’ performance and retrieve visual signs of early detection and deterioration. Further exploitation of this dataset, along with other semantically annotated monitoring sources of the project (gait analysis, cognitive assessment tests, etc.), will open new avenues in Active and Healthy Ageing [67] as well as health monitoring per se as it will provide new insights of the exergames role as assessment tools. This is in line with the emerging trend of knowledge extraction from multimodal sources, towards knowledge understanding by combining information with different granularity ranging from muscles and exercices to cognitive functionality and health status [68].

### Contribution to healthcare

This work aims at semantically enriching exergaming data, thereby enabling semantic processing in the field of exergames. Semantic description of game results alongside open access enables the biomedical semantics community to develop automatic processing, analysis and visualization tools for these data. Such tools are much meaningful in the biomedical domain since further research and study of the produced information will hopefully contribute to a better understanding of any patient’s health state and condition. Abiding to the holistic definition of health by WHO [69] as well as current ehealth emphasis on personalized, citizen driven electronic health record systems (EHRs) [70, 71], it would be reasonable to envision expansion and exploitation of herein presented achievements within future EHR systems. This may be merely attempted by incorporating or even better “semantically linking” a person’s EHR with exergaming behavior and performance data. The advantages of such an EHR approach promoting joint exploitation of heterogeneous information sources [72], would be twofold. First, the EHR information would give a better understanding of the exergaming information and its value in the context of the general health. Secondly, the EHR information, decoded to exergaming aspects, will lead to automatic, design user-tailored exercise protocols, taking into account any possible conditions and limitations.

#### The need for evaluation of exergames

Several studies highlight the importance of exergames as monitoring and evaluation tools which is achieved through the collection and analysis of data produced by their metrics [9, 10, 16] and the gaming environment [73]. However, the absence of large volumes of research that evaluates these data hinders the full integration of exergames in healthcare systems [44]. This work enhances the prospects for further research on evaluating exergames and opens up new ways for open linked game data, thereby facilitating their analysis, verification and understanding by the scientific community.

#### The need for comparison of game’s metrics and algorithms to external monitoring devices

As mentioned previously in this document, some serious games integrate interfaces performing various measurements through game controllers such as energy expenditure. Hence, there is a need to compare their results with data from corresponding measurements from external devices as well as self-monitoring devices in terms of quantified self. To this end, several studies indicate that these measurements should be published as open data enabling researchers to compare them and verify their reliability [16]. In the exergame ontology, these measurements are treated as they were data produced by game metrics and described using the same format, laying the basis for publication of such data.

#### Real-time data analysis

In the context of collecting data in home environments, there is an increasing interest in automated analysis. Data produced in such environments can be analyzed in real time, reducing the costs of health services [74]. Health professionals can provide advice to patients based on decision support systems that consume these data automatically, thereby facilitating personalized healthcare [75, 76].

#### Decentralized information

Moreover, the need to store data in a central database is reduced since patients can play games at their homes which results in the development of a decentralized knowledge base. For instance, information concerning games, exercises and game results could be stored in different locations and recovered through queries over multiple SPARQL Endpoints. Linking patient data from multiple sources may result in a better understanding of health-related problems and even in acquisition of new knowledge. The latter can only be achieved though, under the condition that the data will be open. By opening access to medical databases we can save valuable time facilitating early detection of diseases [77]. For instance, datasets from open public health initiative [78], in which federal health agencies publish health-related data along with population data can be linked with data produced by exergames. Moreover, they can be combined with or even extend patient personal health records derived from well-known personal health record systems such as Microsoft HealthVault, Dossia and World Medical Card [79].

#### Unobtrusive monitoring

Nowadays, a noteworthy number of research efforts is carried out on early detection of cognitive decline utilizing sensors at home [80–82], paying extra attention in preserving a sense of unobtrusiveness. Serious games have just been considered as potential unobtrusive monitoring sources [30]. Therefore, ontologies like the one proposed in this paper will further facilitate the evolution of the serious games into considerable sources of information.

### Challenges

#### Big data

The advent of embedding processors to everyday devices, converting them to smart objects, opens the road towards ubiquitous computing [83]. Quantified-self, including data about diet, mood, physical activity, sleep quality etc., is leveraged from such technological advances, as they facilitate acquisition of daily living data. Smart sensors, self-reporting via mobile applications and other devices are employed to the quantified-self realization [84], resulting in large amounts of data. Big data has been receiving an increased attention in biomedical and healthcare applications, paving research from hypothesis-driven to data-driven. Therefore, there is an increasing demand in new analysis techniques development, following the size, variety and complexity of these data hindering information retrieval [85, 86]. Big data are either formed as structured data, such as those produced by games, or unstructured information. The unstructured data constitute more than 80 percent of the available data [86]. Considering the ever increasing use of serious games, the data produced is expected to scale exponentially. Taking into account the rapid increase in the volume of these data, standards for the exchange and linkage should be developed by health professionals, so as to facilitate data interoperability and integration on different platforms [75]. Furthermore, special attention should be paid to the storage and distribution limitations as well as the development of techniques, tools and infrastructures for data processing and analysis [74].

#### Privacy

Despite the large number of positive statements about the need for transparency on information about clinical trials over many years [87, 88], as far as privacy is concerned, disclosing users’ personal data should be carefully handled [15]. In alignment with the patient commons, it must be ensured that any information system used in storage would protect them from unauthorized access, since such data are sensitive while ownership issues are not always clear [74]. In the realm of data openness, special attention should be paid when publishing game results on the web to ensure the protection of privacy [15]. In this study, information about game sessions was published obscuring any information that may reveal whose data are included in each session. Additionally, the protocol followed by the pilots of this work has been approved by the Bioethics Committee of the Medical School of the Aristotle University of Thessaloniki (No.93/26-6-2014). However, continuous identification of users to different sources is a risk that increases with the volume of data [89]. In addition, apart from the raw data, origin, type and acquisition method should be part of the metadata schema. On the other hand, sharing data that is incomplete and not described properly may have an adverse effect, thereby leading to incorrect assumptions [90].

### Weaknesses and limitations

Although the power of data openness and sharing is acknowledged, there are some concerns in the scientific community about publishing data on the web. More specifically, many researchers are reluctant to open their data as they are afraid that others will not follow the same approach or that they could not be able to control data access [90]. In recent years, there have been efforts contributing to this direction by reinforcing data access and exchange policies. In 2010, U.S. President’s Council of Advisors on Science and Technology (PCAST) issued a report [91] about the potential of health information technology to improve healthcare, underlining the need for the adaptation of a universal exchange language for healthcare information. Other studies state that RDF can be served as a Universal Healthcare Exchange Language, since it is format independent and all existing vocabularies such as Health Level 7 (HL7) and Clinical Document Architecture (CDA) can be mapped to RDF [92].

With regard to the exergame ontology, a limitation is that this study focused mainly on the high-level concepts’ description such as game controllers, goals, game metrics and presentation hardware, while existing ontologies offer a more detailed description of a game itself (e.g. game scenario and game entities). Moreover, the ontology includes only structured exercises, which is going to be addressed in the authors’ future work by incorporating physical activity as well. Furthermore, possible weaknesses of users such as vision and movement problems as well as mental disorders are not part of the player’s description. For instance, the results produced during a game session may vary between a healthy user and a user with mobility problems. The authors intend to study corresponding ontologies and integrate them in the exergame ontology.

Finally, this work included a small number of real patients and exercises focusing on establishing the basis correctly and studying the feasibility of the employed infrastructure. Performance and scalability evaluation with a large number of seniors, participating in a complete protocol (in terms of exercises) will take place within a newly funded research project (www.uncap.eu) where this infrastructure will be further exploited.

### Future work

#### Linking game results with other datasets

The next decade will be the beginning of integrated gaming, when game data and social network data will be linked with other datasets [4] such as electronic health records [44]. An attempt to this direction was made by Aetna, a US insurance company that developed an open platform facilitating data integration from various sources such as medical records [84]. Game data can be combined with data produced by smart phones in the context of utilizing sensors such as accelerometers and GPS towards producing large amounts of location specific data concerning physical activity and physical exercise [86]. Apart from fitness-related data, information from other sources can also be combined with games such as data about diet, sleep quality and allergies. The need for such data integration has led to the emergence of Data as a Service (DaaS) platforms such as “linked life data” that provides access to data from various biomedical databases through a single SPARQL Endpoint [93]. With the aid of these technologies, anonymous data can be effortlessly extracted and utilized by pharmaceutical and insurance companies as well as agencies on health related information to improve drug design and health protocols altogether. Towards this direction, we aim to semantically describe all exergames that are part of the web FitForall platform. Additionally and apart from the instances developed in this work representing contemporary game controllers that are already linked to DBpedia, other concrete entities will be linked to existing datasets as well. More specifically, game metrics, exercises involved in games as well as ailments for which exercises might be employed as treatments or preventative measures will be linked to DBpedia and relevant datasets which utilize biomedical ontologies such as SNOMED and ICD. Moreover, when the amount of RDF triples representing game sessions and results grows, the number of links with other LOD datasets will be increased as well and as a result, exergame-related datasets could then become part of the LOD cloud.

#### Game analytics and semantic processing tools

Apart from linking game results with other datasets, there is an increasing interest in game analytics in the field of game research. Players today have a wide range of ages, backgrounds, intellectual abilities and motivations. The need for understanding player’s behavior and experience during the game has led to integration of game analytics into design and development process [94]. Data from game metrics and player’s behavior are collected and analyzed throughout the game, revealing valuable information that range from details about the players’ profile to design-related information such as software bugs and design problems [95]. Game analytics constitute an important field of business intelligence for the game industry, while the combination of game data with other information sets such as market reports and quality assurance systems facilitates knowledge management, marketing and decision making. The semantic description of games, facilitates searching and retrieval of their data and enables the development of knowledge extraction and data mining tools through automatic processes and semantic web crawlers, opening the way for serious game and exergame analytics.

#### Personalized exercise protocols

Today, the rapidly increasing gaming population, regardless of age and background, has led to the maximization of the need for customized games [27]. In the context of physical activity and exercise, recommendation systems for exergames may be developed, utilizing automated reasoning. In this manner, personalized exercise protocols can be designed, taking into account several factors such as game sessions data, biological factors as well as possible disabilities.

## Conclusions

In this work, a unified model for the semantic representation of exergames is proposed, while actual data from game sessions are published as open data on the web. Aligning with a commons approach, this study aims to be the outset of an exergame commons initiative, which will establish shared standards across the researchers and facilitate exergame research. Last but not least, this exergame commons and open exergame data initiative is one of the very first efforts to enable the concept of Open Trials (an initiative that will aggregate information from a wide variety of existing sources in order to provide a comprehensive picture of the data and documents related to all trials of medicines and other treatments around the world) for health monitoring, in general, as well as, Active and Healthy Ageing more specifically.

## Abbreviations

CAC: Controller application communication
CDA: Clinical document architecture
DGO: Digital game ontology
EHR: Electronic health record
FOAF: Friend of a friend
GCM: Game content model
GOP: Game ontology project
HL7: Health level 7
ICD: International classification of diseases
LOD: Linked open data
LODE: Live OWL documentation environment
MMSE: Mini-mental state examination
OPA: Ontology of physical activity
OPE: Ontology of physical exercise
OWL: Web ontology language
PCAST: President’s Council of Advisors on Science and Technology
PURL: Persistent uniform; resource locator
RDF: Resource description framework
RDFS: Resource description framework schema
SNOMED: Systematized nomenclature of medicine
SWRL: Semantic web rule language
URI: Uniform resource identifier
wFFA: Web fitforall
