# Erratum to: Towards exergaming commons: composing the exergame ontology for publishing open game data

**DOI:** 10.1186/s13326-016-0082-0

**Published:** 2016-06-02

**Authors:** Giorgos Bamparopoulos, Evdokimos Konstantinidis, Charalampos Bratsas, Panagiotis D. Bamidis

**Affiliations:** Medical Physics Laboratory, Medical School, Faculty of Health Sciences, Aristotle University of Thessaloniki, Thessaloniki, Greece; Mathematics Department, Aristotle University of Thessaloniki, Thessaloniki, Greece

After publication of the original article [1] it was brought to our attention that an acknowledgement was missing from the article. The authors would therefore like to add the following acknowledgement, and offer their apologies that this was missed out in the original publication:

This work was supported in part by the European Union’s Seventh Framework Programme (Project USEFIL, GA 288532; http://www.usefil.eu), as well as the LLM Care (www.llmcare.gr) self-funded initiative that emerged as the business exploitation of the Long Lasting Memories (LLM Project) (www.longlastingmemories.eu) originally funded by the ICT-CIP-PSP Programme.
